# Effects of *Platycladus orientalis* Leaf Extract on the Growth Performance, Fur-Production, Serum Parameters, and Intestinal Microbiota of Raccoon Dogs

**DOI:** 10.3390/ani13193151

**Published:** 2023-10-09

**Authors:** Xiao Li, Xiaoli Chen, Weitao Yuan, Xiuli Zhang, Aipeng Mao, Weigang Zhao, Naiquan Yao, Xuming Deng, Chao Xu

**Affiliations:** 1 Institute of Special Animal and Plant Sciences, Chinese Academy of Agricultural Sciences, 4899 Juye Street, Changchun 130112, China; lix9805@163.com (X.L.);; 2 Innovation Center for Feeding and Utilization of Special Animals in Jinlin Province and Research Center for Microbial Feed Engineering of Special Animals in Jilin Province, 4899 Juye Street, Changchun 130112, China; 3 College of Veterinary Medicine, Jilin University, Changchun 130062, China; xiuli23@jlu.edu.cn (X.Z.); dengxm@jlu.edu.cn (X.D.); 4 College of Animal Science and Technology, Jilin Agricultural University, Changchun 130118, China

**Keywords:** *Platycladus orientalis* leaf extract, performance, nutrient digestibility and metabolism, serum biochemistry, intestinal microbiota, raccoon dog

## Abstract

**Simple Summary:**

The raccoon dog is one of the most important fur-producing animals. In recent years, due to the gradual ban on the use of antibiotics as growth promoters in various countries, the cost of raccoon dog farming has risen. Plant extracts are considered potential alternative products because they are completely natural, cost-effective, and abundant in various biologically active ingredients. *Platycladus orientalis* leaves are rich in diverse bioactive components such as flavonoids and polysaccharides, which possess high medical and nutritional value. In the present study, raccoon dogs were fed diets containing *P. orientalis* leaf extract (PLE), and their growth performance, fur quality, serum parameters, and intestinal microbiota were examined. The results indicated that supplementation with appropriate PLE enhanced growth performance and fur quality while also promoting the intestinal health of raccoon dogs. This research highlights the promising potential of PLE as a valuable feed additive for fur animal production.

**Abstract:**

*Platycladus orientalis* leaves are rich in flavonoids and polysaccharides, which offer high medicinal and nutritional benefits. This study aimed to investigate the impact of *P. orientalis* leaf extract (PLE) on the growth performance, fur quality, serum parameters, and intestinal microbiota of raccoon dogs. Sixty healthy male black raccoon dogs, aged 85 (±5) days, were randomly assigned to four groups and fed a basal diet supplemented with 0, 0.25, 0.50, and 1.00 g/kg PLE for 125 days (designated as groups P0, P1, P2, and P3, respectively). The results revealed that the raccoon dogs in group P1 exhibited increased average daily gain and underfur length while showing a decreased feed/gain ratio compared to group P0 (*p* < 0.05). However, the heart index in group P2 was significantly lower than in group P0 (*p* < 0.05), and the kidney index and serum alanine aminotransferase activities in group P3 were higher than in groups P2 and P0 (*p* < 0.05), suggesting potential adverse effects at higher PLE dosages. Notably, dietary PLE supplementation led to a reduction in serum glucose concentrations (*p* < 0.05), which may have implications for glucose regulation. Furthermore, the study explored the impact of dietary supplementation with 0.25 g/kg PLE on the raccoon dogs’ intestinal microbiota using high-throughput sequencing. The results showed significant alterations in the microbial community structure, with a notable decrease in the abundance of *Prevotella copri* in response to 0.25 g/kg PLE supplementation (*p* < 0.05). In conclusion, supplementing raccoon dogs’ diet with 0.25 g/kg PLE can lead to improved growth performance and a positive influence on the intestinal microbiota. However, caution should be exercised regarding higher dosages, as they may have adverse effects on certain parameters. As a result, PLE holds promise as a potential feed additive for fur animal production.

## 1. Introduction

The raccoon dog (*Nyctereutes procyonoides*) is a medium-sized omnivorous animal that belongs to the order Carnivora and the family Canidae. Originally native to East Asia, this species was introduced into the European region in the 1950s, primarily for fur production [1]. Antibiotics have long been extensively employed in livestock production. Nevertheless, the excessive use of antibiotics has resulted in a surge of antibiotic-resistant pathogens, posing a significant threat to human health. Recognizing this concern, many countries have prohibited the utilization of antibiotics as growth promoters. However, banning the addition of antibiotics in the feed results in a decrease in the production performance of animals, which leads to an increase in production costs. Consequently, there is now an intensified imperative to explore alternative approaches to antibiotics within the realm of raccoon dog production. Plant extracts are natural, green, safe, and highly effective biologically active substances extracted from natural plants. It was reported that plant extracts can regulate immune function, improve performance, and intestinal health [2,3], which can be effective substitutes for antibiotics.

*Platycladus orientalis* (L.) Franco is an evergreen tree belonging to the genus *Platycladus Spach* of the family Cupressaceae. It has a wide distribution around the world, including regions such as China, Korea, Japan, and others. *P. orientalis* leaves are the dried needles of *P. orientalis* (L.) Franco and have been widely used in therapeutic medications since ancient times. The major biologically active components of *P. orientalis* leaves include flavonoids, polysaccharides, and tannins. Pharmacological studies focusing on *P. orientalis* leaves revealed their multiple biological effects, including anti-inflammatory, antibacterial, antioxidant, and hair growth-promoting activities [4]. Fan et al. conducted a study on the anti-inflammatory properties of *P. orientalis* leaves both in vivo and in vitro and discovered that the ethanolic extract of *P. orientalis* leaves contained anti-inflammatory compounds that had a beneficial effect on the production of NO and TNF-α [5]. Another study found that the water extract of *P. orientalis* leaves could promote hair growth, enhance hair follicle construction, increase skin thickness, and expand hair bulb diameter in a dose-dependent manner [6]. Flavonoids in *P. orientalis* leaves include quercetin, and it was reported that quercetin significantly increased the activities of superoxide dismutase and glutathione peroxidase while decreasing catalase activity and malondialdehyde content in the serum of laying hens [7]. Although *P. orientalis* leaves are generally considered to have low toxicity, the prolonged oral administration of higher doses was found to have significant effects on growth, liver function, and blood parameters in rats [4]. The abundant and cheap resource of *P. orientalis* leaves, along with their beneficial health effects and potential for promoting hair growth, makes them a promising candidate for use in the fur industry. However, there are limited studies on the application of *P. orientalis* leaves in fur animals, and no prior reports focused on their use in raccoon dogs. Therefore, this study aimed to investigate the effects of dietary PLE on raccoon dogs’ growth performance, fur quality, serum parameters, and intestinal microflora. The purpose of this study was to explore the impact of PLE on the raccoon dogs’ diet, determine the optimal addition level, and provide a theoretical basis for the application of PLE in fur animal production.

## 2. Materials and Methods

### 2.1. Experimental Animal Design

The experiment was conducted at the fur animal production base of the Institute of Special Animal and Plant Sciences, Chinese Academy of Agricultural Sciences. A total of sixty male black raccoon dogs, aged 85 (±5) days, with an initial body weight of 3.08 ± 0.42 kg (mean ± SD), were randomly assigned to four dietary treatments. Each treatment consisted of fifteen replicates of one raccoon dog. The dietary treatments were as follows: P0 (basal diet); P1 (basal diet + 0.25 g/kg PLE); P2 (basal diet + 0.50 g/kg PLE); and P3 (basal diet + 1.00 g/kg PLE). According to the recommended therapeutic dosages of *P. orientalis* leaves and its extracts for several common poultry and livestock diseases, as well as the concentrated ratio of PLE in this experiment, the prophylactic dosage was predicted based on the body weight and daily feed intake of the raccoon dogs. Finally, the appropriate addition level of approximately 0.50 g/kg was determined. Consequently, the doses of 0.25, 0.50, and 1.00 g/kg were selected. The experiment lasted for 125 days, and the composition and nutritional level of the experimental diets are shown in Table 1. The PLE provided in this experiment was premixed into the powdered chow. The powdered chow was thoroughly mixed with water in a 1:5 ratio and then allowed to stand for half an hour to form a semisolid diet before feeding. Throughout the entire trial period, all animals were housed individually in separate cages and were fed twice a day at 7:00 and 14:00. They had free access to water to ensure adequate hydration.

The PLE used in this study was purchased from Lanzhou Wotelaisi Biotechnology Co., Ltd (Lanzhou, China). The preparation process for PLE was as follows: *P. orientalis* leaves were crushed and decocted three times using 70% ethanol at temperatures of 64–65 °C for 2 h each time. Subsequently, the extract was filtered, concentrated under reduced pressure, and then spray-dried at temperatures of 70–75 °C. Finally, after being pulverized and passing through a 100-mesh sieve, the ultimate extract was obtained. The flavonoids were determined using the aluminum trichloride method [8]. A standard curve was constructed using rutin as the standard, resulting in a regression equation (y = 0.0125x − 0.0055, R^2^ = 0.9992). The polysaccharides were determined using the phenol–sulfuric acid method [9]. A standard curve was established using glucose as the standard, yielding a regression equation (y = 3.825x + 0.0009, R^2^ = 0.9978). The tannins were determined following the guidelines outlined in the Chinese Pharmacopoeia 2020 (Part four), Appendix 2202, using gallic acid as the standard [10]. This resulted in a regression equation (y = 0.0111x + 0.0021, R^2^ = 0.9983). The PLE had a flavonoid content of 18.92%, a polysaccharide content of 6.34%, and a tannin content of 1.03%.

### 2.2. Growth Performance and Digestion Metabolism Trail

On day 0 and day 125, the initial and final body weights of the raccoon dogs were recorded separately before the morning feeding, and the daily feed intake was also recorded. The body weight and feed consumption data were used to calculate the average daily gain, average daily feed intake, and feed/gain ratio.

A 3-day digestion and metabolism trial was conducted in the middle of the test. For this trial, eight healthy raccoon dogs with similar body weights were selected from each group. At the end of the trial, the feces and urine were collected separately and then mixed. To fix the nitrogen, an appropriate amount of 10% sulfuric acid was added. The nutrient content in the feces and nitrogen content in the urine were determined and calculated using the method described by Wang et al. [2].

### 2.3. Visceral Index, Fur Quality, and Measurement of Serum Parameters

At the end of the trial, eight raccoon dogs per group were randomly selected, and blood was collected from the hind limb vein in the morning after fasting using 5 mL vacuum blood collection tubes without any additives. The blood was then centrifuged at 3500 rpm for 15 min at 4 °C and stored at −80 °C for the analysis of serum parameters. Serum aspartate aminotransferase (AST), alanine aminotransferase (ALT), alkaline phosphatase (ALP), total protein (TP), albumin (ALB), urea, and glucose (GLU) levels were measured using commercial colorimetric kits purchased from Beijing Zhongsheng Beikong Biochemistry Company (Beijing, China) and analyzed on the Beckman AU480 automatic biochemistry analyzer (Vitalab Selectra E, Spankeren, The Netherlands). Serum total antioxidant capacity (T-AOC), superoxide dismutase (SOD), and malondialdehyde (MDA) were determined using commercial kits purchased from Nanjing Jiancheng Bioengineering Institute (Nanjing, China). Serum immunoglobulin A (IgA), immunoglobulin G (IgG), and immunoglobulin M (IgM) levels were measured using ELISA kits purchased from Shanghai Jianglai Biotechnology Co., Ltd (Shanghai, China).

Subsequently, the raccoon dogs were humanely slaughtered via electric shock according to the guidelines outlined in the Welfare of Animals Kept for Fur Production. The colon contents were collected using sterile centrifuge tubes and stored at −80 °C for the analysis of the intestinal microbiota. Visceral organs, including the heart, liver, spleen, and kidney were excised and weighed. The weight and length of the skin were recorded after fleshing and drying. The length of guard hair and underfur from the dorsal surface of the mesothorax was measured with a modified micrometer. Fur quality was graded on a scale ranging from 1 (poorest) to 10 (best).

### 2.4. Sequencing and Analysis of Intestinal Microbiota

The DNA from the colon intestinal contents was extracted using the Magnetic Soil and Stool DNA Kit (TianGen, Beijing, China). The V3-V4 region of the 16S rRNA gene was amplified using primers 341F (5′-CCTAYGGGRBGCASCAG-3′) and 806R (5′-GGACTACNNGGGTATCTAAT-3′). Sequencing libraries were generated using the NEB Next^®^ Ultra™ II FS DNA PCR-free Library Prep Kit (New England Biolabs, Ipswich, MA, USA) and then quantified using Qubit and Real-time Quantitative Polymerase Chain Reaction. Subsequently, the libraries were sequenced on an Illumina NovaSeq 6000 platform, generating 250 bp paired-end reads.

The raw tags were obtained by merging paired-end reads using FLASH (V1.2.11) software. Quality filtering of the raw tags was performed using the fastp (version 0.23.1) software. The tags were then compared with the Silva database using the UCHIME Algorithm to detect chimera sequences, which were subsequently removed, leaving the effective tags. These effective tags were denoised using DADA2 in the QIIME2 software (Version QIIME2-202006) to obtain initial Amplicon Sequence Variants (ASVs), and ASVs with an abundance of less than 5 were filtered out. Species annotation was carried out using the QIIME2 software and the Silva Database.

The Chao1, Pielou_e, Shannon, and Simpson indices were calculated using the QIIME2 software. For PCoA analysis plots, the ggplot2, extrafont, grid, and ade4 packages of R software (version 3.5.3) were utilized. Differences in community structure between groups were analyzed using Adonis functions in the QIIME2 software. Additionally, the T-test analysis in R software was used to identify significantly different communities at the phylum and species levels.

### 2.5. Statistical Analysis

The statistical analysis was performed using SPSS 26.0 software (IBM SPSS Statistics Version 26.0, IBM Corp., Armonk, NY, USA). The Shapiro–Wilk normality test and normal Q-Q plots were used for the normality test. The statistical data processing was performed using a one-way analysis of variance (one-way ANOVA). Differences between groups were assessed using Tukey’s test. The results are presented as the mean and standard deviation (mean ± SD). A significance level of *p* < 0.05 was considered statistically significant.

## 3. Results

### 3.1. Growth Performance and Fur Quality

The effects of PLE on growth performance and fur quality in raccoon dogs are presented in Table 2. At the end of the experiment, there was no significant difference in the final body weight and average daily feed intake among the groups (*p* > 0.05). However, the average daily gain in group P1 significantly increases, while the feed/gain ratio decreases compared to group P0 (*p* < 0.05).

The PLE supplement has no significant effect on skin length, skin weight, and fur score (*p* > 0.05). On the other hand, guard hair length significantly increases in group P3 compared to group P2 (*p* < 0.05). The underfur length in group P1 is significantly higher than in group P0 (*p* < 0.05).

### 3.2. Nutrient Digestibility and Nitrogen Metabolism

Table 3 and Table 4 present the effects of PLE on nutrient digestibility and nitrogen metabolism in raccoon dogs. There are no significant differences observed in the apparent digestibility of dry matter, crude protein, and crude fat among the groups (*p* > 0.05). Similarly, no significant differences are found in nitrogen intake, fecal nitrogen, urine nitrogen, nitrogen retention, net protein utilization rate, and protein biological value among the groups (*p* > 0.05). 

### 3.3. Viscera Index

Table 5 shows the effects of PLE on the viscera index of raccoon dogs. Compared with group P0, the heart index in group P2 significantly decreases (*p* < 0.05). The liver index in group P3 significantly increases when compared to both group P0 and group P2 (*p* < 0.05). There is no significant difference in the spleen and kidney index among the groups (*p* > 0.05).

### 3.4. Serum Parameters

The effects of different dietary PLE levels on the serum parameters of raccoon dogs are shown in Table 6. There is no significant difference among AST, ALP, TP, ALB, and Urea (*p* > 0.05). The activity of ALT in group P3 is significantly higher than that in group P2 and group P0 (*p* < 0.05). In comparison to group P0, the addition of PLE at varying concentrations to the diet significantly reduces serum GLU levels (*p* < 0.05). No significant differences are found in T-AOC, SOD, MDA, IgA, IgG, and IgM among the groups (*p* > 0.05).

### 3.5. Intestinal Microbial Composition Analysis

As shown in Table S1, after quality filtering, a total of 1,425,461 reads are produced, with a mean sequence number of 89,091 ± 16,317 reads per sample. Goods_coverage for all samples is over 99.9%, indicating sufficient sequencing depth (Table S2). 

**Figure 1:**
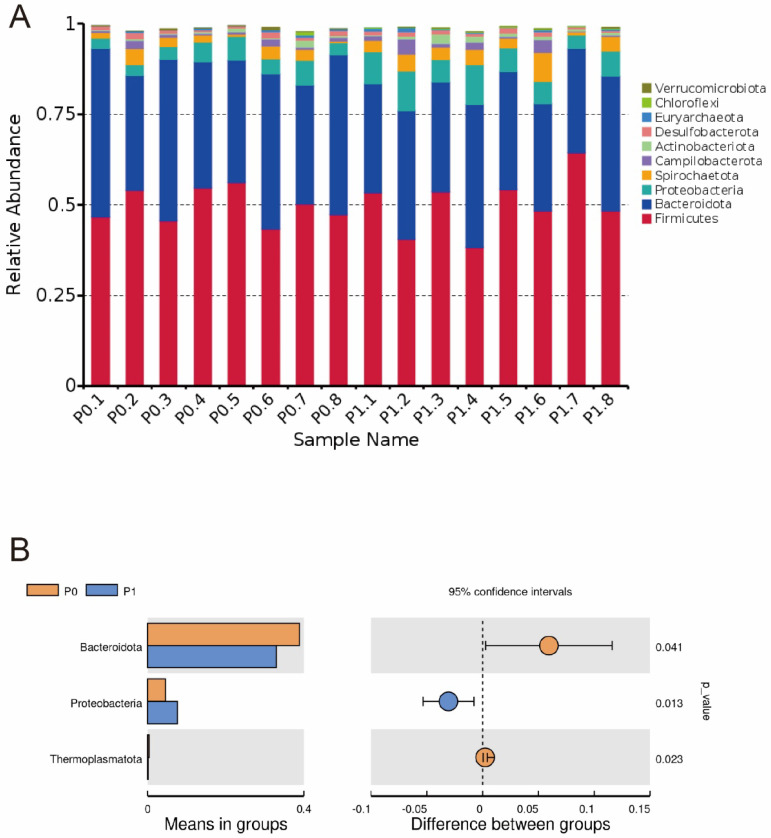
Relative abundance of the top 10 phyla in the intestinal microbiota (**A**). T-test analysis of intestinal microbiota composition at the phylum level (**B**). Note: the P0.1 to P0.8 represent the samples from group P0, and the P1.1 to P1.8 represent the samples from group P1. Dietary treatment: P0 (basal diet); P1 (basal diet + 0.25 g/kg PLE).

**Figure 2:**
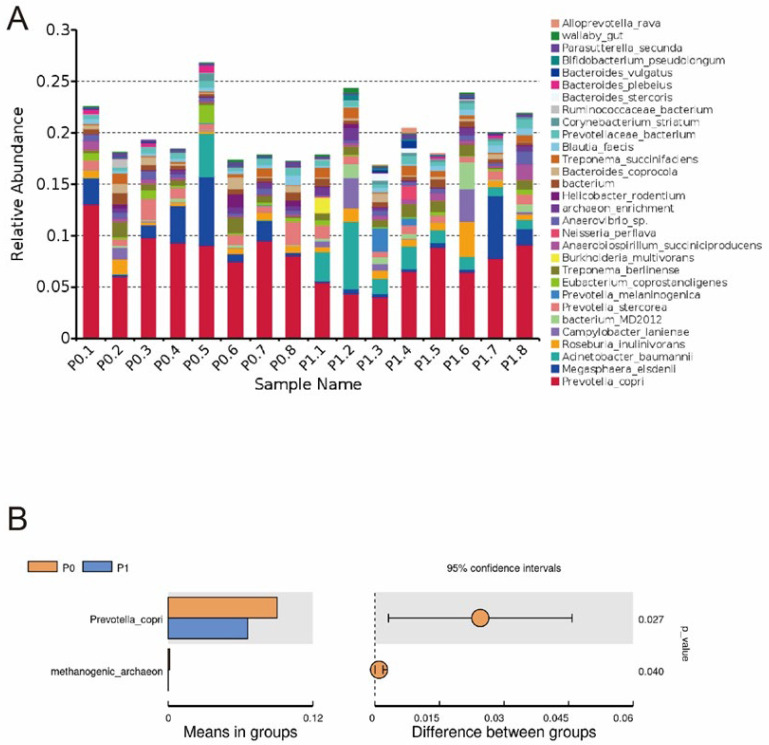
Relative abundance of the top 30 species in the intestinal microbiota (**A**). T-test analysis of intestinal microbiota composition at the species level (**B**). Note: the P0.1 to P0.8 represent the samples from group P0, and the P1.1 to P1.8 represent the samples from group P1. Dietary treatment: P0 (basal diet); P1 (basal diet + 0.25 g/kg PLE).

At the phylum level, the top 10 bacteria in relative abundance are identified, and the results indicate that Firmicutes, Bacteroidota, and Proteobacteria are the most abundant bacteria in the colon contents (Figure 1A). The relative abundance of Bacteroidota and Thermoplasmatota decreases, while Proteobacteria increases in group P1 compared to group P0 (Figure 1B, *p* < 0.05). At the species level, the dominant microbiota is *Prevotella copri* (Figure 2A). Supplementing with PLE decreases the relative abundance of *P. copri* (Figure 2B, *p* < 0.05).

**Figure 3:**
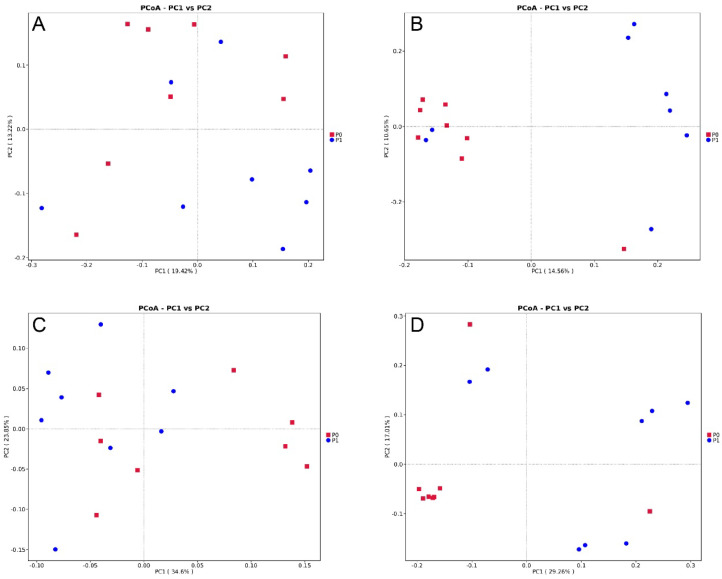
Comparisons of the bacterial communities in the intestinal of raccoon dogs. Principal coordinate analyses (PCoA) based on Bray-Curtis distance (**A**), Jaccard (**B**), weighted UniFrac distance (**C**), and unweighted UniFrac distances (**D**). Dietary treatment: P0 (basal diet); P1 (basal diet + 0.25 g/kg PLE).

Table S2 shows the alpha diversity indices of the bacterial communities of raccoon dogs. The results show that the differences in the observed OTUs, Chao1, Pielou_e, Shannon, and Simpson indices between the two groups are not significant (*p* > 0.05). The PCoA (Figure 3) and Adonis analysis (Table 7) show that the bacteria composition of group P1 is significantly separated from that of group P0 based on Jaccard, weighted UniFrac distance, and unweighted UniFrac distance matrices (Adonis: *p* < 0.05).

## 4. Discussion

Raccoon dogs are fur-bearing animals, and their fur quality is closely associated with economic benefits [2]. *P. orientalis* (L.) Franco is an evergreen tree with abundant resources and a wide distribution. Its extract contains flavonoids, polysaccharides, and tannins. PLE exhibits diverse biological activities, including anti-inflammatory, antioxidant, and hair growth-promoting activities. However, there have been few reports to date on the effects of PLE on fur-producing animals. Therefore, this study evaluated the impact of PLE on raccoon dogs’ growth performance, serum parameters, and intestinal microbiota.

Plant extracts have been extensively researched within the context of livestock and poultry, with compelling evidence of their ability to enhance animal performance [11,12]. For example, Chen et al. [13] observed that supplementing the diet of weaned piglets with *Broussonetia papyrifera* leaf extract resulted in an increased final weight and a reduced feed conversion ratio. Similarly, the inclusion of 0.3 g/kg of *Eucommia ulmoides* leaf extract in the diet improved the activities of duodenal and jejunal digestive enzymes and intestinal morphology, leading to a significant increase in the average daily gain of weaned piglets [14]. Our results are similar to these studies, as supplementing raccoon dogs’ daily diet with 0.25 g/kg of PLE leads to a significant increase in their average daily gain while reducing the feed/gain ratio. This effect may be attributed to the presence of flavonoids and polysaccharides within PLE. It is reported that plant flavonoids and polysaccharides contribute to the improvement in intestinal morphology, resulting in improved nutrient absorption [15,16]. Furthermore, these compounds have the capacity to promote the proliferation of beneficial intestinal bacteria while suppressing the growth of pathogenic counterparts, thereby enhancing growth performance [17,18]. Additionally, plant flavonoids and polysaccharides were shown to elevate the levels of growth factors such as growth hormone and insulin-like growth factor-1 [19,20]. All of these factors possibly contribute to the promotion of raccoon dogs’ growth by PLE. However, further investigation is needed to explore the specific mechanism.

This study also finds that the inclusion of 0.25 g/kg PLE in raccoon dogs’ feed results in enhanced underfur length, leading to an improvement in fur quality. The hair growth-promoting activities of *P. orientalis* leaves have been recorded since ancient times and validated through modern pharmacological research [4]. Ahn et al. found that the mixture of the ethanol extract of *P. orientalis* leaves and α-terpineol could increase insulin-like growth factor-1, vascular endothelial growth factor, and cell proliferation through the upregulation of wnt3 and β-catenin expressions [21]. Another report demonstrated that the hot water extract of *P. orientalis* leaves, containing kaempferol and isoquercetin, could promote hair growth by inducing the anagen phase of hair follicles in C57BL/6N mice [22]. In addition, the presence of flavonoids such as quercetin and quercitrin [23,24] in *P. orientalis* leaves was shown to function in hair growth. Quercetin could stimulate resting hair follicles to grow through rapid follicular keratinocyte proliferation and replenishment of perifollicular microvasculature [25], while quercitrin could stimulate hair growth by enhancing cellular energy metabolism and increasing the production of growth factors via activation of the MAPK/CREB signaling pathway [26]. These findings suggest that the effect of PLE on improving raccoon dogs’ fur quality may result from a combination of various bioactive compounds present in PLE.

Nutrient apparent digestibility reflects the absorption of nutrients in animals, while nitrogen metabolism indicates their efficiency in utilizing proteins. Previous studies have demonstrated that plant extracts can enhance the digestion, absorption, and utilization of dietary nutrients [27,28]. However, some studies have found no improvement in nutrient utilization despite supplementing diets with plant extracts [29,30]. This discrepancy could be attributed to variations in the bioactive components present in different plant extracts. In our study, we investigate the effects of feeding PLE to raccoon dogs on the apparent digestibility of nutrients and nitrogen metabolism, and we find that PLE has no significant impact on these parameters. However, when we supplement the raccoon dogs’ diets with 0.25 g/kg of PLE, we observe an improvement in protein utilization. Interestingly, as the level of PLE supplementation increases, the protein utilization in raccoon dogs is hindered. These findings indicate that a suitable dose of PLE can positively impact protein utilization in raccoon dogs. Plant extracts are rich in bioactive compounds that can enhance nutrient digestion and absorption by improving the histological structure of the intestines and modifying the composition of intestinal microbiota [31,32]. However, it is important to note that some plant extracts contain higher levels of anti-nutrients, such as tannins, phenolic acids, and gossypol, which might exert adverse effects on nutrient utilization in animals. PLE contains a certain level of tannins, which can form stable complexes with proteins, starches, and digestive enzymes, thereby preventing the proper absorption and utilization of nutrients [33]. Therefore, we speculate that the decrease in protein utilization in raccoon dogs with high doses of PLE may be attributed to the increased levels of tannins.

The visceral index can directly reflect the health status of the body. Generally, a higher heart index indicates better cardiac ejection function, implying superior heart function. An elevated liver index usually points to liver damage, with drug-induced liver toxicity being a common cause of such injury [34]. Similarly, the rising kidney index suggests potential renomegaly. In our research, we discovered that compared to the control group, adding 0.50 g/kg of PLE to the diet significantly reduces the heart index of the raccoon dogs, while adding 1.00 g/kg of PLE significantly increases the liver index of the raccoon dogs. Moreover, as the level of PLE in the diet increases, there is also a trend of an increased kidney index in the raccoon dogs. This implies that prolonged high-dose PLE feeding may weaken the raccoon dogs’ hearts, cause liver damage, and elevate the risk of renomegaly. This may be related to the drug toxicity caused by the long-term administration of a high dose of PLE [4].

The spleen plays a vital role as one of the main immune organs, serving as the center for both cellular immunity and humoral immunity in raccoon dogs. An elevation within the normal physiological range of the spleen index indicates better functioning. Studies have reported significant anti-inflammatory effects of *P. orientalis* leaf flavonoids on lipopolysaccharide-induced RAW 264.7 mouse macrophage cells, leading to the inhibition of NO, IL-6, and TNF-α secretion through the suppression of inflammatory-related gene expressions [35]. Additionally, *P. orientalis* leaf polyphenols were also shown to decrease lipopolysaccharide-induced overproduction of TNF-α, Pro-1L-1β, and IL-6, as well as their protein expression in THP-1 cells [24]. This highlights the potential of PLE to inhibit inflammation and enhance the body’s immunity. However, our results reveal that PLE has no effect on the spleen index of raccoon dogs, and similar findings are observed in raccoon dogs’ serum IgA, IgG, and IgM levels. This could be attributed to the fact that the raccoon dogs had reached adulthood and were in a healthy condition.

Many studies have found that flavonoids and polyphenols in *P. orientalis* exhibit antioxidant activities [36,37]. The polyphenols in *P. orientalis* leaves were shown to have scavenging activity against DPPH, ABTS, hydroxyl radicals, and superoxide anions [24]. Moreover, the ethanol extract of *P. orientalis* leaves could effectively inhibit the formation of MDA during lipid peroxidation [38]. Although the study observes some improvements in the activity of serum T-AOC and MDA contents when 0.25 g/kg of PLE is added to raccoon dogs’ feed, the effect is not statistically significant. This may be possibly related to the contents of polyphenols, flavonoids, and other natural antioxidant active substances present in PLE, as the extraction of bioactive compounds from *P. orientalis* leaves is significantly influenced by solvent polarity [39].

The concentrations of TP, ALB, and Urea in the serum reflect the metabolic status of protein in the body to a certain extent [40]. In this study, there was no significant difference in serum TP, ALB, and Urea among the groups. Serum levels of ALT and AST are sensitive biomarkers used for evaluating liver function, and their elevated serum levels indicate liver damage [41]. Our findings reveal that the addition of PLE at levels of 0.25 or 0.50 g/kg in the diet has no impact on serum ALT and AST levels in raccoon dogs. However, when PLE is added at a level of 1.00 g/kg, it leads to elevated serum ALT and AST levels in raccoon dogs, which align with the results of the liver index. This suggests that long-term high-dose feeding of PLE may possess potential hepatotoxicity and can cause liver damage in raccoon dogs. In addition, our research reveals that incorporating PLE into the raccoon dogs’ diet leads to a significant reduction in fasting blood glucose levels. This positive effect can be attributed to the presence of apigenin in *P. orientalis* leaves [42]. Apigenin may potentially decrease blood glucose levels by inhibiting the activities of α-amylase and α-glucosidase [43]. However, the specific mechanism needs further investigation due to the complex composition of PLE.

Animal intestinal microbiota plays an important role in regulating host metabolism, maintaining animal health, improving body resistance, and preventing pathogen colonization [44,45,46]. Research has found that the inclusion of plant extracts in diets can enhance host metabolism and disease resistance by regulating the host’s intestinal microbiota [47]. In the current study, we observe that diet supplementation with 0.25 g/kg PLE influences the composition of microflora in raccoon dogs’ intestines. The abundance of Bacteroidota and Thermoplasmatota in group P1 significantly decreased, while Proteobacteria increased compared to group P0 at the phylum level. Furthermore, at the species level, the abundance of *P. copri* and methanogenic archaeon in group P1 shows a significant decrease. *P. copri* was associated with various inflammatory diseases [48], as it encodes superoxide reductase and phosphoadenosine phosphosulfate reductase, which might promote inflammation development [49]. A study conducted in vitro has shown that *P. copri* can stimulate the production of pro-inflammatory cytokines such as IL-6, IL-23, and IL-17, which are associated with Th17 immune responses [50]. *P. copri* was also linked to body weight loss and increased epithelial inflammation in a mouse model of colitis [51]. Moreover, it induces insulin resistance by elevating circulating concentrations of branched-chain amino acids [52]. This result suggests that intestinal microbes may be collectively involved in the regulation of raccoon dogs’ serum glucose levels. Overall, these findings suggest that the addition of 0.25 g/kg PLE to the diet may have improved raccoon dogs’ intestinal health and enhanced their resistance to disease by inhibiting the growth of *P. copri*. 

## 5. Conclusions

The study demonstrates that dietary supplementation with 0.25 g/kg PLE increases the average daily gain and underfur length while decreasing the feed/gain ratio of raccoon dogs. Moreover, the dietary supplement with 0.25 g/kg PLE can influence host metabolism and health by regulating the composition of the intestinal microbiota. It is worth noting that exercising caution is essential when considering higher PLE dosages, as they may have adverse effects on the health of raccoon dogs. In conclusion, PLE shows promise as a valuable feed additive for fur animal production.

## Figures and Tables

**Table 1:** Composition and nutrient levels of the basal diet.

Items	Content (%)
**Ingredients**	
Extruded corn	39.0
Soybean meal	20.0
Distillers dried grains with solubles	10.0
Corn germ meal	12.0
Fish meal	4.00
Meat meal	2.00
Extruded soybean meal	2.00
Hemoglobin power	2.00
Lysine	0.75
Methionine	0.25
Soybean oil	4.00
Calcium hydrogen phosphate	1.50
Calcium carbonate	1.50
Salt	0.20
Premix ^1^	0.80
Total	100
**Nutrient levels (air-dry basis) ^2^**	
Gross energy/(MJ/kg)	18.4
Crude Protein	24.4
Ether Extract	7.70
Ash	7.72
Calcium	1.22
Phosphorus	1.34

^1^ The premix provided the following per kilogram of the diet: VA 2500 IU; VD3 400 IU; VE 24 IU; VB1 5 mg; VB2 3.6 mg; VK3 0.8 mg; antioxidant 0.04 mg; VB6 3 mg; VB12 0.009 mg; biotin 0.004 mg; folic acid 0.2 mg; nicotinic 10 mg; calcium pantothenate 6.8 mg; VC 25 mg; Cu 7.2 mg; Fe 38.4 mg; Mn 19.2 mg; Zn 31.2 mg; I 0.576 mg; Se 0.12 mg; Co 0.096 mg; choline chloride 360 mg. ^2^ The nutrient levels were measured values.

**Table 2:** Effects of dietary *Platycladus orientalis* (L.) Franco leaf extract (PLE) levels on growth performance and fur quality of raccoon dogs.

Items	P0	P1	P2	P3	*p*-Value
Growth performance					
Initial body weight (kg)	3.08 ± 0.39	3.07 ± 0.49	3.07 ± 0.54	3.08 ± 0.28	1.000
Final body weight (kg)	7.83 ± 0.81	8.38 ± 0.50	7.99 ± 0.53	8.00 ± 0.58	0.10
Average daily feed intake (g/d)	272 ± 0.95	271 ± 1.72	271 ± 1.89	270 ± 2.94	0.25
Average daily gain (g/d)	38.0 ± 4.91 ^b^	42.5 ± 4.53 ^a^	39.3 ± 3.28 ^ab^	39.4 ± 3.72 ^ab^	0.034
Feed/gain ratio	7.28 ± 1.06 ^a^	6.45 ± 0.71 ^b^	6.94 ± 0.58 ^ab^	6.92 ± 0.73 ^ab^	0.048
Fur quality					
Skin length (g)	97.8 ± 4.73	101 ± 2.04	102 ± 3.58	102 ± 4.05	0.10
Skin weight (g)	572 ± 65.8	597 ± 52.7	597 ± 55.0	618 ± 68.7	0.52
Guard hair length (cm)	10.2 ± 0.36 ^ab^	10.4 ± 0.49 ^ab^	9.92 ± 0.42 ^b^	10.7 ± 0.40 ^a^	0.007
Underfur length (cm)	6.74 ± 0.60 ^b^	7.37 ± 0.36 ^a^	6.81 ± 0.28 ^b^	7.10 ± 0.45 ^ab^	0.028
Fur score (score)	7.20 ± 0.55	7.72 ± 0.29	7.34 ± 0.29	7.75 ± 0.45	0.025

Note: different lowercase letters in the same row indicate significant difference (*p* < 0.05); data with the same letter or no letter indicate that the difference is not significant (*p* > 0.05). Dietary treatment: P0 (basal diet); P1 (basal diet + 0.25 g/kg PLE); P2 (basal diet + 0.50 g/kg PLE); P3 (basal diet + 1.00 g/kg PLE).

**Table 3:** Effects of dietary *Platycladus orientalis* (L.) Franco leaf extract (PLE) levels on nutrient digestibility of raccoon dogs.

Items (%)	P0	P1	P2	P3	*p*-Value
Dry matter digestibility	73.5 ± 1.32	74.5 ± 1.60	72.5 ± 2.06	74.2 ± 2.17	0.14
Crude protein digestibility	74.1 ± 1.72	75.4 ± 1.79	74.0 ± 2.49	74.8 ± 3.52	0.67
Crude fat digestibility	83.6 ± 2.24	82.6 ± 3.29	83.5 ± 4.36	86.1 ± 2.52	0.18

Dietary treatment: P0 (basal diet); P1 (basal diet + 0.25 g/kg PLE); P2 (basal diet + 0.50 g/kg PLE); P3 (basal diet + 1.00 g/kg PLE).

**Table 4:** Effects of dietary *Platycladus orientalis* (L.) Franco leaf extract (PLE) levels on nitrogen metabolism of raccoon dogs.

Items	P0	P1	P2	P3	*p*-Value
Nitrogen intake (g/d)	9.87 ± 0.064	9.88 ± 0.064	9.87 ± 0.031	9.87 ± 0.064	0.94
Fecal nitrogen (g/d)	2.40 ± 0.17	2.28 ± 0.18	2.40 ± 0.24	2.34 ± 0.32	0.70
Urine nitrogen (g/d)	4.40 ± 0.46	4.16 ± 0.34	4.16 ± 0.95	4.42 ± 0.54	0.73
Nitrogen retention (g/d)	3.08 ± 0.40	3.44 ± 0.27	3.31 ± 0.99	3.12 ± 0.50	0.60
Net protein utilization rate (%)	31.1 ± 4.02	34.8 ± 2.64	33.5 ± 10.08	31.6 ± 5.02	0.60
Protein biological value (%)	41.2 ± 5.66	45.3 ± 3.90	44.2 ± 12.89	41.4 ± 6.47	0.66

Dietary treatment: P0 (basal diet); P1 (basal diet + 0.25 g/kg PLE); P2 (basal diet + 0.50 g/kg PLE); P3 (basal diet + 1.00 g/kg PLE).

**Table 5:** Effects of dietary *Platycladus orientalis* (L.) Franco leaf extract (PLE) levels on viscera index of raccoon dogs.

Items (%)	P0	P1	P2	P3	*p*-Value
Heart index	0.47 ± 0.038 ^a^	0.43 ± 0.025 ^ab^	0.40 ± 0.032 ^b^	0.44 ± 0.043 ^ab^	0.012
Liver index	3.54 ± 0.31 ^b^	3.63 ± 0.37 ^ab^	3.61 ± 0.45 ^b^	4.16 ± 0.44 ^a^	0.014
Spleen index	0.16 ± 0.067	0.15 ± 0.035	0.13 ± 0.018	0.16 ± 0.033	0.63
Kidney index	0.53 ± 0.048	0.57 ± 0.041	0.58 ± 0.056	0.59 ± 0.081	0.15

Note: different lowercase letters in the same row indicate significant difference (*p* < 0.05); data with the same letter or no letter indicate that the difference is not significant (*p* > 0.05). Dietary treatment: P0 (basal diet); P1 (basal diet + 0.25 g/kg PLE); P2 (basal diet + 0.50 g/kg PLE); P3 (basal diet + 1.00 g/kg PLE).

**Table 6:** Effects of dietary *Platycladus orientalis* (L.) Franco leaf extract (PLE) levels on serum parameters of raccoon dogs.

Items	P0	P1	P2	P3	*p*-Value
Serum biochemical index					
AST (U/L)	46.4 ± 11.8	53.0 ± 9.01	54.8 ± 11.2	62.3 ± 13.4	0.073
ALT (U/L)	14.9 ± 3.97 ^b^	18.5 ± 4.42 ^ab^	17.1 ± 4.58 ^b^	23.5 ± 4.42 ^a^	0.004
ALP (U/L)	32.8 ± 13.0	30.2 ± 19.4	36.1 ± 15.9	35.7 ± 16.8	0.88
TP (g/L)	53.2 ± 0.80	52.9 ± 4.45	53.8 ± 2.41	53.5 ± 2.04	0.93
ALB (g/L)	33.1 ± 2.15	30.7 ± 5.49	33.2 ± 4.14	33.3 ± 2.34	0.46
Urea (mmol/L)	7.08 ± 1.58	6.82 ± 1.78	7.38 ± 1.04	7.16 ± 0.94	0.88
GLU (mmol/L)	5.73 ± 0.79 ^a^	4.84 ± 0.59 ^b^	4.78 ± 0.42 ^b^	4.54 ± 0.49 ^b^	0.002
Serum antioxidant index					
T-AOC (U/mL)	0.36 ± 0.075	0.40 ± 0.065	0.39 ± 0.059	0.35 ± 0.057	0.42
SOD (U/mL)	10.7 ± 1.18	10.8 ± 0.60	11.1 ± 0.83	10.5 ± 0.52	0.52
MDA (nmol/mL)	6.57 ± 1.12	5.85 ± 1.01	6.19 ± 0.78	6.25 ± 0.53	0.47
Serum immunoglobulin index					
IgA (μg/L)	278 ± 72.7	244 ± 61.0	255 ± 61.2	275 ± 84.6	0.73
IgG (g/L)	2.84 ± 1.08	3.06 ± 1.02	2.67 ± 1.15	2.46 ± 1.24	0.75
IgM (μg/L)	171 ± 44.6	169. ± 24.7	147 ± 31.9	181 ± 34.5	0.27

Note: different lowercase letters in the same row indicate significant difference (*p* < 0.05); data with the same letter or no letter indicate that the difference is not significant (*p* > 0.05). Abbreviations: AST, aspartate aminotransferase; ALT, alanine aminotransferase; ALP, alkaline phosphatase; TP, total protein; ALB, albumin; GLU, glucose. Dietary treatment: P0 (basal diet); P1 (basal diet + 0.25 g/kg PLE); P2 (basal diet + 0.50 g/kg PLE); P3 (basal diet + 1.00 g/kg PLE).

**Table 7:** Adonis analysis of the bacterial communities in the intestinal of raccoon dogs.

Items	R^2^	*p*-Value
Bray–Curtis	0.093	0.063
Jaccard	0.098	0.010
Weighted UniFrac	0.15	0.028
Unweighted UniFrac	0.17	0.001

## Data Availability

The data that support the findings of this study are available from the corresponding author upon reasonable request.

## Supplementary Materials

The following supporting information can be downloaded at https://www.mdpi.com/article/10.3390/ani13193151/s1. Table S1: Statistical Results of Date Processing of the Bacteria in the Intestinal of Raccoon Dogs; Table S2: Alpha Diversity Indices of the Bacteria in the Intestinal of Raccoon Dogs.
