# Supplementary material for: Effects of Platycladus orientalis Leaf Extract on the Growth Performance, Fur-Production, Serum Parameters, and Intestinal Microbiota of Raccoon Dogs
Source: Animals (Basel). 2023 Oct 9;13(19):3151. doi: 10.3390/ani13193151 (PMC10571531; doi:10.3390/ani13193151)
Supplement: Supplementary file 1 [file animals-13-03151-s001.zip › animals-2576785-supplementary.pdf]

animals

MDPI

**Table S1.** Statistical Results of Date Processing of the Bacteria in the Intestinal of Raccoon Dogs.

| Samples | RawPE | Combined | Qualified | Nochime | Base(nt) | Avglen(nt) | GC | Q20 | Q30 | Effective% |
|---|---|---|---|---|---|---|---|---|---|---|
| P0.1 | 124847 | 123015 | 119831 | 110013 | 45949784 | 417.68 | 52.53% | 97.43% | 92.27% | 88.12% |
| P0.2 | 106494 | 104908 | 102273 | 92622 | 38489438 | 415.55 | 52.60% | 97.40% | 92.24% | 86.97% |
| P0.3 | 94062 | 92703 | 90315 | 82964 | 34621252 | 417.3 | 52.45% | 97.44% | 92.32% | 88.20% |
| P0.4 | 79304 | 78035 | 76038 | 70582 | 29582186 | 419.12 | 52.50% | 97.28% | 91.98% | 89.00% |
| P0.5 | 90149 | 88729 | 86358 | 79323 | 33305165 | 419.87 | 52.48% | 97.32% | 92.01% | 87.99% |
| P0.6 | 93675 | 92319 | 90022 | 82499 | 34497936 | 418.16 | 52.58% | 97.47% | 92.33% | 88.07% |
| P0.7 | 114363 | 112562 | 109647 | 97596 | 40770650 | 417.75 | 52.96% | 97.43% | 92.28% | 85.34% |
| P0.8 | 101948 | 100529 | 98085 | 89510 | 37297794 | 416.69 | 52.57% | 97.41% | 92.24% | 87.80% |
| P1.1 | 85407 | 84227 | 82230 | 75657 | 31479744 | 416.09 | 52.62% | 97.43% | 92.28% | 88.58% |
| P1.2 | 72680 | 71713 | 70062 | 63751 | 26646086 | 417.97 | 52.75% | 97.54% | 92.46% | 87.71% |
| P1.3 | 134126 | 132144 | 128150 | 110072 | 45905960 | 417.05 | 52.64% | 97.39% | 92.21% | 82.07% |
| P1.4 | 132409 | 130207 | 126343 | 111315 | 46595590 | 418.59 | 52.65% | 97.30% | 91.96% | 84.07% |
| P1.5 | 130527 | 128501 | 124893 | 116009 | 48295010 | 416.3 | 52.75% | 97.43% | 92.27% | 88.88% |
| P1.6 | 112941 | 111140 | 107853 | 98936 | 41200909 | 416.44 | 52.65% | 97.33% | 92.06% | 87.60% |
| P1.7 | 88546 | 86999 | 84277 | 78502 | 32927035 | 419.44 | 52.77% | 97.21% | 91.82% | 88.66% |
| P1.8 | 74919 | 73679 | 71525 | 66110 | 27659072 | 418.38 | 52.44% | 97.22% | 91.85% | 88.24% |
| | | | | 1,425,461 | | | | | | |
| | | | | 89,091 | | | | | | |
| | | | | 16,317 | | | | | | |

Note: the P0.1 to P0.8 represent the samples from group P0, and the P1.1 to P1.8 represent the samples from group P1. Dietary treatment: P0 (basal diet); P1 (basal diet + 0.25 g/kg PLE)

**Table S2.** Alpha Diversity Indices of the Bacteria in the Intestinal of Raccoon Dogs.

| Samples | OTU | Chao1 | Pielou_e | Shannon | Simpson | Goods_ coverage |
|---|---|---|---|---|---|---|
| P0.1 | 986 | 998.2791 | 0.8426 | 8.3805 | 0.993 | 0.9994 |
| P0.2 | 1004 | 1005.32 | 0.8694 | 8.6688 | 0.9954 | 0.9998 |
| P0.3 | 834 | 835.875 | 0.8446 | 8.1956 | 0.9926 | 0.9998 |
| P0.4 | 729 | 730.4737 | 0.8147 | 7.7473 | 0.9826 | 0.9999 |
| P0.5 | 712 | 712.8333 | 0.7902 | 7.4875 | 0.9813 | 0.9999 |
| P0.6 | 814 | 815.037 | 0.8566 | 8.2828 | 0.9936 | 0.9999 |
| P0.7 | 1348 | 1353.8879 | 0.8122 | 8.4441 | 0.9909 | 0.9993 |
| P0.8 | 786 | 787.4865 | 0.8291 | 7.9748 | 0.9912 | 0.9998 |
| P1.1 | 796 | 798.3684 | 0.8603 | 8.2905 | 0.9937 | 0.9998 |
| P1.2 | 766 | 766.375 | 0.8363 | 8.0124 | 0.9897 | 0.9999 |
| P1.3 | 1171 | 1193.987 | 0.8439 | 8.6023 | 0.9946 | 0.9989 |
| P1.4 | 1367 | 1379.9655 | 0.8629 | 8.9887 | 0.9959 | 0.9991 |
| P1.5 | 1046 | 1063.9577 | 0.8407 | 8.4327 | 0.994 | 0.9991 |

| | | | | | | |
|---|---|---|---|---|---|---|
| P1.6 | 942 | 955.5227 | 0.8287 | 8.1874 | 0.9918 | 0.9994 |
| P1.7 | 680 | 683.25 | 0.7661 | 7.2085 | 0.976 | 0.9997 |
| P1.8 | 716 | 716 | 0.8382 | 7.9496 | 0.992 | 1 |

Note: the P0.1 to P0.8 represent the samples from group P0, and the P1.1 to P1.8 represent the samples from group P1. Dietary treatment: P0 (basal diet); P1 (basal diet + 0.25 g/kg PLE)
